# Bmi1 regulates self-renewal and epithelial to mesenchymal transition in breast cancer cells through Nanog

**DOI:** 10.1186/1471-2407-14-785

**Published:** 2014-10-28

**Authors:** Anurag N Paranjape, Sai A Balaji, Tamoghna Mandal, Esthelin Vittal Krushik, Pradeep Nagaraj, Geetashree Mukherjee, Annapoorni Rangarajan

**Affiliations:** Department of Molecular Reproduction, Development and Genetics, Indian Institute of Science, Bangalore, 560012 Karnataka India; Department of Pathology, Kidwai Memorial Institute of Oncology, Bangalore, 560029 Karnataka India

**Keywords:** Bmi1, Breast cancer stem cells, Drug-resistance, Epithelial to mesenchymal transition, Nanog, NFκB

## Abstract

**Background:**

The Bmi1 polycomb ring finger oncogene, a transcriptional repressor belonging to the Polycomb group of proteins plays an important role in the regulation of stem cell self-renewal and is elevated in several cancers. In the current study, we have explored the role of Bmi1 in regulating the stemness and drug resistance of breast cancer cells.

**Methods:**

Using real time PCR and immunohistochemistry primary breast tissues were analyzed. Retro- and lentiviruses were utilized to overexpress and knockdown Bmi1, RT-PCR and Western blot was performed to evaluate mRNA and protein expression. Stemness properties were analyzed by flow cytometry and sphere-formation and tumor formation was determined by mouse xenograft experiments. Dual luciferase assay was employed to assess promoter activity and MTT assay was used to analyze drug response.

**Results:**

We found Bmi1 overexpression in 64% of grade III invasive ductal breast adenocarcinomas compared to normal breast tissues. Bmi1 overexpression in immortalized and transformed breast epithelial cells increased their sphere-forming efficiency, induced epithelial to mesenchymal transition (EMT) with an increase in the expression of stemness-related genes. Knockdown of Bmi1 in tumorigenic breast cells induced epithelial morphology, reduced expression of stemness-related genes, decreased the IC_50_ values of doxorubicin and abrogated tumor-formation. Bmi1-high tumors showed elevated Nanog expression whereas the tumors with lower Bmi1 showed reduced Nanog levels. Overexpression of Bmi1 increased Nanog levels whereas knockdown of Bmi1 reduced its expression. Dual luciferase promoter-reporter assay revealed Bmi1 positively regulated the Nanog and NFκB promoter activity. RT-PCR analysis showed that Bmi1 overexpression activated the NFκB pathway whereas Bmi1 knockdown reduced the expression of NFκB target genes, suggesting that Bmi1 might regulate Nanog expression through the NFκB pathway.

**Conclusions:**

Our study showed that Bmi1 is overexpressed in several high-grade, invasive ductal breast adenocarcinomas, thus supporting its role as a prognostic marker. While Bmi1 overexpression increased self-renewal and promoted EMT, its knockdown reversed EMT, reduced stemness, and rendered cells drug sensitive, thus highlighting a crucial role for Bmi1 in regulating the stemness and drug response of breast cancer cells. Bmi1 may control self-renewal through the regulation of Nanog expression via the NFκB pathway.

**Electronic supplementary material:**

The online version of this article (doi:10.1186/1471-2407-14-785) contains supplementary material, which is available to authorized users.

## Background

A growing body of evidence suggests that cancer is organized in a hierarchical fashion exhibiting functional heterogeneity wherein few ‘cancer stem cells’ (CSCs) with stem-like properties drive tumor proliferation and progression [1]. First identified in leukemia [2, 3], such tumor-initiating cells with extensive proliferative potential have now been identified in several solid tumors [4], such as gliomas [5], pancreatic cancers [6], colon cancers [7], and breast carcinomas [8]. CSCs have also been found to be inherently drug-resistant [9], thus making it difficult to target them, and thereby thought to contribute to cancer relapse. Interestingly, cancer cells undergoing epithelial to mesenchymal transition, considered to be a pre-requisite for solid tumor metastasis, have been shown to acquire stem-like properties [10]. Further, we have recently shown that the very transcription factors that bring about an EMT also lead to an increased expression of ABC family of transporters, thereby increasing drug resistance [11]. Thus, these data suggest that the properties of self-renewal, EMT, and drug resistance may all be linked [12]. Therefore, understanding the pathways and mechanisms that regulate the stem-like properties of cancer cells is fundamental for their effective therapeutic targeting.

Bmi1 belongs to the Polycomb Group (PcG) gene family of proteins that function as chromatin modifiers and play important roles in stem cell maintenance as well as cancer development [13]. Bmi1 was first identified as a c-Myc co-operating oncoprotein inducing B or T cell leukemia [14]. Since then, aberrant overexpression of Bmi1 has been detected in several human cancers including lymphoma, acute myeloid leukemia, colorectal carcinoma, liver carcinoma, non-small cell lung cancer, breast carcinoma, prostate cancer, head and neck squamous cell carcinoma, medulloblastoma, and glioblastoma [14–23]. Significantly, elevated Bmi1 expression has been associated with poor prognosis in several cancers including breast carcinomas [24], highlighting the prognostic relevance of Bmi1 expression.

Bmi1 serves as the key regulatory component of the PRC1 complex (Polycomb repressive complex 1) which modulates chromatin structure, thereby regulating gene transcription [25]. It has been shown to regulate the expression of the Ink4a locus which encodes for two tumor suppressor proteins p16Ink4a and p14Arf, thereby regulating cell proliferation and senescence [21]. However, Bmi1 has been shown to play a role in tumorigenesis in Ink4A-deficient models [25], suggesting that it may regulate other genes important in cancer. Consistent with this, Bmi1 has been shown to repress tumor suppressor PTEN [26], induce telomerase [17], activate Akt/GSK3β/Snail pathway [16], and cooperate with Twist to repress E-cadherin [15]. In the current study we have investigated the effects of Bmi1 overexpression and down-modulation on the expression of genes that regulate EMT and stemness, and further explored the molecular mechanisms downstream of Bmi1 that regulate EMT and stem cell properties in breast cancer cells.

Recent studies have revealed a role for Bmi1 in the regulation of self-renewal in both normal and cancer stem cells. For example, Bmi1 was found to be necessary for the self-renewal of normal hematopoietic stem cells as well as leukemic stem and progenitor cells [27, 28]. It has also been implicated in the regulation of self-renewal of neural stem cells [29] as well as glioma stem cells [30]. Bmi1 has also been shown to regulate self-renewal and proliferation of cancer stem cells from other tumor types such as hepatocellular carcinoma, prostate cancer, and pancreatic cancer [31–33]. In the context of breast tissue, it was found that in both normal and malignant mammary stem cells, Hedgehog signaling regulates self-renewal through Bmi1 [34]. Yet, the stemness genes regulated by Bmi1 remain unknown. Thus, understanding the molecular mechanisms by which Bmi1 regulates the stem cell properties of cancer cells is likely to pave the way for newer therapeutic modules.

In the present study we have analyzed the status of Bmi1 expression at mRNA and protein levels in Indian patients with grade III invasive ductal breast adenocarcinomas. Further, we assessed the effects of Bmi1 overexpression and shRNA-mediated knockdown on stemness, self-renewal, EMT and drug-resistance of breast cancer cells. Our study supports the observations that Bmi1 could be a potential prognostic marker in breast cancer. Further, we show that mechanistically, Bmi1 may regulate stemness by positively regulating Nanog expression through the NFκB pathway.

## Methods

### Collection of normal and cancerous breast tissue

Normal and cancerous breast tissues were procured from Kidwai Memorial Institute of Oncology (KMIO) Bangalore, in accordance with the Institutional Review Board and in compliance with the ethical guidelines of KMIO and the Indian Institute of Science. Patient consent was acquired in a written form before the surgery. The normal tissue was excised ~6 cm away from the tumor and was confirmed by pathologists for absence of tumor cells. For RNA isolation, normal and tumor tissue chunks were collected in RNAlater (Qiagen, Hilden, Germany). The paraffin blocks for normal and tumor tissues were also obtained from KMIO. The tissues were sectioned using microtome and were fixed on glass slides for further staining and analysis.

### Immunohistochemistry

Immunohistochemical staining was carried out as described previously [35]. Briefly, the paraffin embedded tissue sections were deparaffinized with xylene and were rehydrated. 5% hydrogen peroxide was used to quench the peroxidase activity. For antigen retrieval the sections were cooked under high pressure by placing the sections in 10 mM sodium citrate buffer (pH 6) in a pressure cooker. Sections were blocked with 4% non-fat dry milk, incubated overnight with primary antibodies [Bmi1, Nanog, (Santa Cruz Biotechnology, Santa Cruz, CA, USA), CD44 (Cell Signaling Technology, Beverly, MA, USA)] at 4°C. The sections were washed and stained with secondary anti-mouse and anti-goat antibodies (Vector labs, Burlingame, CA, USA) on the following day and detected using ABC color development kit (Vector labs). The immunohistochemical intensity was semi-quantitatively scored by an experienced pathologist based on the intensity of the Bmi1 staining as described previously [35]. Highest intensity was graded ‘high’ (+++), moderate intensity was graded ‘medium’ (++), and lowest intensity was graded as ‘low’ (+).

### RNA isolation, RT-PCR, and real time PCR

Using motorized homogenizer, the snap frozen tissue (~100 mg) was ground and total RNA was isolated using Tri-reagent (Sigma Aldrich, St Louis, MO, USA) according to manufacturer’s protocol. cDNA was synthesized from 1 μg of total RNA using Gene-Amp RNA PCR cDNA synthesis kit (Applied Biosystems, Carlsbad, CA, USA). Primers were designed using Primer3 online tool. HPRT, β2-microglobulin, or RPL were used as normalizing controls. Sequence of primers used is provided in Additional file 1: Figure S1. Real Time PCR was performed according to manufacturer’s protocol using DyNAmo SYBR Green qPCR Kit (Finnzymes, Vantaa, Finland) with ROX passive reference dye using Applied Biosystem’s 7900 HT Real Time PCR system.

### Cell culture, virus production, and infection

HEK293T cells, breast cancer epithelial cells MCF7 and MDAMB231 (ATCC), and derived cells were cultured in DMEM with 10% fetal bovine serum. NBLE and NBLE-derived cells were cultured as described previously [36] in DMEM-F12 with growth factors (10 ng/ml hEGF, 1 mg/ml hydrocortisone, 10 mg/ml insulin, 4 ng/ml heparin) (Sigma Aldrich) and B27 (Invitrogen, Carlsbad, CA, USA). HMLE and HMLE-Bmi1 cells were cultured in DMEM-F12 media with 10 ng/ml hEGF, 0.5 μg/ml hydrocortisone, and 10 μg/ml insulin (Sigma Aldrich). All media also included penicillin (1 kU/ml) and streptomycin (0.1 mg/ml). Using WI siRNA selection program the siRNA against Bmi1 was designed [37] (Additional file 1: Figure S2). These custom shRNA oligos were purchased from Sigma Aldrich and were cloned into pLKO1 vector as described previously [38]. The control vectors, pBABEpuro-Bmi1, and pLKO1-shBmi1 were transfected along with packaging plasmids (pUMVC3 or pHR’D8.2 and pCMV-VSVG) into HEK293T cells using Fugene-6 (Roche, Mannheim, Germany) according to manufacturer’s protocol. After 48 hrs viral supernatant was harvested and filtered through 0.45 μm filters. Target cells were infected along with 4 μg/ml protamine sulfate and 0.1 M HEPES buffer (Sigma Aldrich) for 6 hrs in 37°C incubator. After 48 hrs of infection the cells were drug selected with 1 μg/ml puromycin.

### Flow cytometry analysis

The cells were trypsinized and were incubated in 37°C incubator for 60 min for surface antigen recovery, and stained with CD44-PE and CD24-FITC, or CD44-PE-Cy7 and CD24-AF610 (BD Biosciences) for 45 min at 4°C in dark. Stained cells were washed twice with PBS and were analyzed in BD FACS-ARIA II (BD Biosciences, San Jose, CA, USA). Unstained cells, CD44-alone and CD24-alone stained cells served as controls.

### Self-renewal assay

For assessing the sphere-forming efficiency, trypsinized cells (5×10^4^) were seeded in 6 well ultra-low attachment plates (Corning) in DMEM-F12 media containing 1% methyl cellulose along with above mentioned growth factors. Sphere size and number was measured after 7 days of seeding.

### Dual luciferase assay

1×10^4^ HEK293T cells were seeded in a 24-well plate and transfected with 800 ng pGL3-Nanog promoter-luciferase plasmid or NFκB promoter-luciferase plasmid, along with 800 ng pLKO1 control vector, or 800 ng of pBABEpuro-Bmi1, or 800 ng pLKO1-shBmi1 plasmids on the following day. The cells were co-transfected with 50 ng of pRLTK plasmid for normalizing. After 48 hrs, luciferase activity was measured using dual-luciferase assay kit (Promega, Madison, WI, USA) using a scintillation counter for 10 sec. Firefly luciferase activity was expressed as relative light units (RLUs) compared to Renilla luciferase activity. pGL3 basic vector was used as negative control and pGL3 control vector was used as positive control in the experiment.

### MTT-based cytotoxicity assay

MTT assay was performed in triplicates in 96-well plates (Greiner Bio-One, Frickenhausen, Germany). After 12 hrs of seeding, various concentrations of doxorubicin were added and the cells were incubated for another 48 hrs. MTT (5 mg/ml) reagent (Sigma Aldrich) was added to each well and the plate was incubated for 4 hrs until the formazan crystals were formed. Crystals were dissolved in DMSO and the plate was read using ELISA reader at 570 nm. Cell viability was expressed as percentage of the absorbance of drug-treated cells, relative to that of the untreated controls.

### *In vivo*tumor formation assay

Animal experiments were performed with approval from Institutional Animal Ethics Committee, IISc. Cells were injected subcutaneously into the flanks of 4–6 week old female nude mice. Tumor size and weight were monitored regularly.

### Immunoblot analysis

Cell lysates were prepared using lysis buffer with 1% NP40 detergent, 0.5% sodium deoxycholate, 0.1% SDS, 50 mM sodium fluoride, 1 mM sodium orthovandate, 10 mM sodium pyrophosphate (Sigma Aldrich) and protease inhibitors (Roche). Protein was quantified with Bradford reagent and equal amount of protein was resolved by SDS-PAGE using Bio-Rad apparatus, transferred to PVDF membrane (Millipore, Billerica, MA, USA) and probed with appropriate antibodies. HRP-coupled secondary antibodies were obtained from Jackson ImmunoResearch (West Grove, PA, USA), and immunoblots were visualized using Pico reagent (Pierce, IL, USA). Following primary antibodies were used: Nanog, ABCC1 (Santa Cruz Biotechnology), N-cadherin (Epitomics, Burlingame, CA, USA), Bmi1 (Cell Signaling Technologies). Anti-α-tubulin antibody (Calbiochem, Darmstadt, Germany) was used as the loading control in all Western blots.

## Results

### Bmi1 is overexpressed in breast cancer tissues

**Figure 1:**
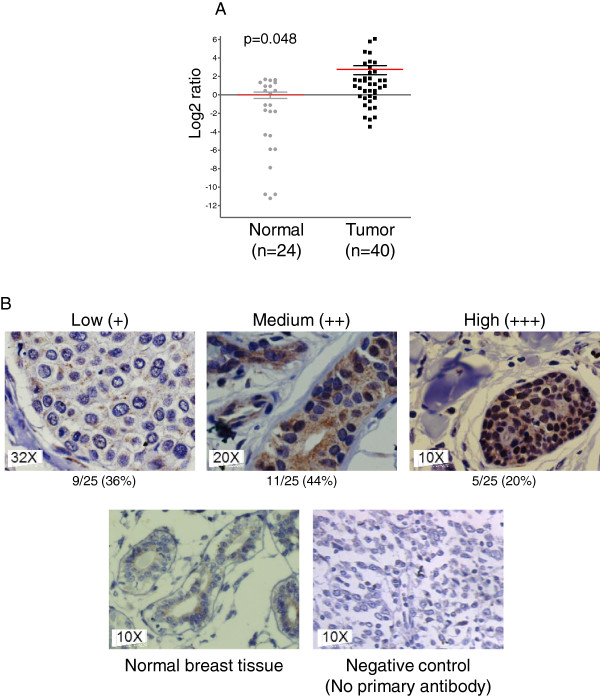
**Bmi1 is overexpressed in breast tumor tissues. A**. Scatter plot shows gene expression of Bmi1 in normal (n =24) and tumorigenic (n =40) breast tissues assessed by qPCR. Log2- ratios for each dot represents data for one sample. Overexpression of Bmi1 mRNA in breast cancer, as compared to normal breast tissue, showed a median change (log2) of 2.75 folds. Statistical significance was calculated using unpaired *t*-test (p =0.0481). **B**. The images show immunohistochemical analysis for Bmi1 in normal and tumor breast tissues. Lower panel shows negative control where primary antibody was excluded.

Previous studies indicated that Bmi1 is overexpressed in various cancers including breast cancer [39]. To investigate Bmi1 expression in Indian breast cancer patient samples, we undertook quantitative real time PCR (qPCR) and immunohistochemistry (IHC) based analyses in primary breast cancer samples that were predominantly grade III invasive ductal breast adenocarcinomas. Analysis by qPCR revealed significant overexpression of Bmi1 in tumor samples, compared to normal tissue (p =0.0481), with a median change (log2) of 2.75 folds (Figure 1A).

Immunohistochemical analysis was performed on 25 breast tumor tissue sections to determine the expression of Bmi1 at protein level. We observed that 64% of the tumors showed presence of Bmi1 protein which varied from low, moderate to high expression (Figure 1B and Additional file 1: Figure S3). Normal breast tissues either lacked Bmi1 or showed lower cytoplasmic expression (Figure 1B). These results indicated that compared to normal breast tissue, Bmi1 expression is higher in breast tumor tissues both at mRNA and protein levels. Our data thus supports the observation [24] that Bmi1 expression could serve as a prognostic marker in breast cancer.

### Overexpression of Bmi1 in immortalized and transformed breast cells increases expression of stemness regulating genes and mesenchymal properties

**Figure 2:**
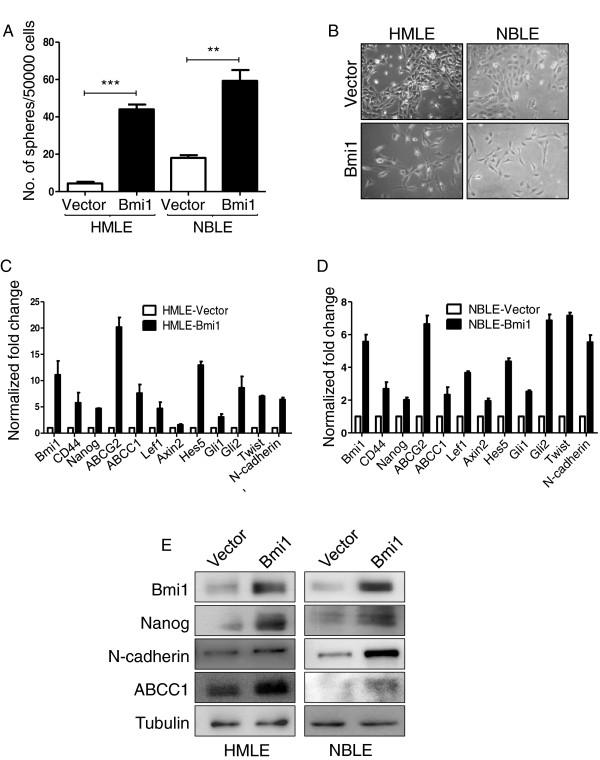
**Overexpression of Bmi1 in breast cells increases stemness and induces EMT. A**. The graph shows number of mammospheres formed in methylcellulose by HMLE and NBLE cells with vector alone control or with Bmi1 overexpression (n =3; error bars indicate s.d, statistical significance was calculated using unpaired *t*-test between number of mammospheres formed by vector control and Bmi1 over expression, ** = p <0.01, *** = p <0.001). **B**. The pictographs show morphology of HMLE and NBLE cells with vector alone control and with Bmi1 overexpression. Graphs showing fold changes (normalized to β2M mRNA levels) of stemness and EMT-related genes in HMLE cells **(C)** and NBLE cells **(D)** with vector alone control (white bars) and with Bmi1 overexpression (black bars), n =3; error bars indicate s.d. **E**. Immunoblot analysis for expression of stemness and EMT related markers in HMLE and NBLE cells with vector alone control and Bmi1 overexpression.

Bmi1 has been shown to be necessary for the self-renewal of normal and malignant breast epithelial cells [34]. However, the effect of Bmi1 on expression of various stemness-related genes has not been studied adequately. Therefore, we overexpressed Bmi1 using retroviral vector (pBABEpuro-Bmi1) in *in vitro* immortalized HMLE [40] and *in vitro* transformed NBLE cells [36] (Figure 2E) and assessed the effect on various aspects of stemness. Mammosphere formation has been used as a measure of *in vitro* stem-like properties [41]. We observed that in HMLE cells that exhibit very low stem cell properties [10] Bmi1 overexpression led to an increase in the number of mammospheres (Figure 2A). In NBLE cells that already exhibited stem cell properties, Bmi1 overexpression further enhanced sphere-formation (Figure 2A). Together these data revealed that Bmi1 enhances the self-renewal potential of mammary epithelial cells.

Since acquisition of stemness has been linked to EMT properties [10], we next gauged the EMT properties of Bmi1 overexpressing cells. Phenotypically, compared to the control empty vector carrying HMLE cells which were largely clusters of epithelial-like cells, the cells overexpressing Bmi1 were more scattered and mesenchymal in appearance (Figure 2B-left panel). NBLE cells that were moderately mesenchymal further acquired long and slender mesenchymal morphology upon Bmi1 overexpression (Figure 2B-right panel). We assessed the status of stemness and EMT-related genes in Bmi1 overexpressing cells by semi-quantitative RT-PCR analysis. We observed that Bmi1 overexpression in HMLE and NBLE cells led to an increase in the expression of stemness related genes such as Nanog, CD44, ABCC1 and ABCG2, downstream effector genes of pathways regulating self-renewal such as Lef1, Axin2, Hes5, Gli1 and Gli2, and EMT-related genes such as Twist and N-cadherin (Figure 2C and D). Further, immunoblot analysis confirmed increased expression of Nanog, N-cadherin, and ABCC1 in these cells upon Bmi1 overexpression (Figure 2E). Taken together, these data revealed that Bmi1 regulates expression of stemness, self-renewal and EMT-related genes, suggesting that Bmi1 may play an important role in inducing stemness properties in mammary epithelial cells. These data are consistent with a previous report showing Hedgehog signaling and Bmi1 playing a crucial role in regulating self-renewal of human mammary stem cells [34].

### Knockdown of Bmi1 in breast cancer cells reduces stemness and induces epithelial morphology

**Figure 3:**
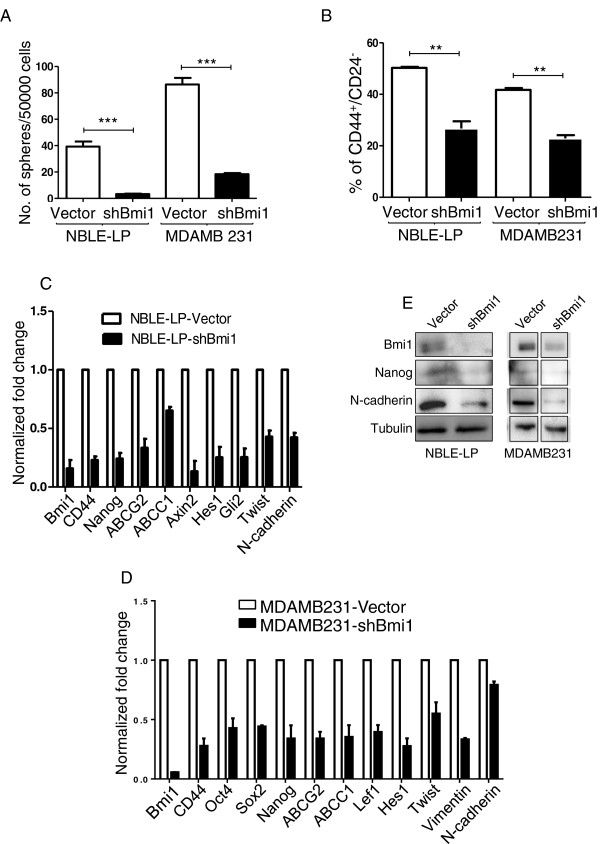
**Knockdown of Bmi1 in breast cancer cells reduces stemness and induces epithelial morphology. A**. The graph shows number of mammospheres formed in methylcellulose by NBLE-LP and MDAMB231 cells with vector alone control or with shBmi1 (n =3; error bars indicate s.d, statistical significance was calculated using unpaired *t*-test between number of mammospheres formed by vector control and Bmi1 knockdown, ** = p <0.01, *** = p <0.001). **B**. The graph shows percentage of CD44^+^/CD24^−^ fraction in NBLE-LP and MDAMB231 cells with vector alone control or with shBmi1 (n =3; error bars indicate s.d, statistical significance was calculated using unpaired *t*-test between percentage of CD44^+^/CD24^−^ in vector control and cells with Bmi1 knockdown, ** = p <0.01, *** = p <0.001). The graphs show fold change normalized to β2M mRNA levels in NBLE-LP **(C)** and MDAMB231 cells **(D)** with vector alone control or with shBmi1, (n =3; error bars indicate s.d). **E**. Immunoblot analysis for stemness and EMT related markers in NBLE-LP and MDAMB231 cells with vector alone control and shBmi1 (MDAMB231 samples were run on the same gel).

We observed that Bmi1 overexpression led to increased expression of stemness, self-renewal, and EMT related genes. To corroborate the specificity of our observations, we undertook shRNA-mediated knockdown of Bmi1 in breast cancer cell lines with higher Bmi1 expression and investigated the effects on stemness properties. We had previously reported that repeated sub-culturing of the *in vitro* transformed NBLE cells led to the generation of late passage cells (NBLE-LP) which show higher expression of Bmi1 compared to the parental cells [36]. Thus, we chose NBLE-LP cells and the invasive MDAMB231 cells for studying the effects of Bmi1 knockdown. For this, we generated a suitable shBmi1 construct in pLKO1 vector. Western blot analysis confirmed effective knockdown of Bmi1 (as shown in Figure 3E). Further, Bmi1 knockdown impaired sphere-formation in both NBLE-LP and MDAMB231 cells (Figure 3A). The CD44^+^/CD24^−^ marker status has been associated with breast cancer stem cell-phenotype [8]. We observed that Bmi1 knockdown reduced the CD44^+^/CD24^−^ fraction compared to control cells (Figure 3B). We also observed that knockdown of Bmi1 in NBLE-LP and MDAMB231 cells resulted in the acquisition of epithelial cobblestone morphology (Additional file 1: Figure S4). Additionally, in NBLE-LP and MDAMB231 cells, we observed that upon Bmi1 knockdown, stemness, self-renewal, and EMT related genes showed reduced expression (Figure 3C and D). Immunoblot analysis further confirmed reduction of Nanog and N-cadherin levels upon Bmi1 knockdown (Figure 3E). These results clearly indicated that Bmi1 plays a crucial role in regulating the stemness properties of breast cancer cells.

### Knockdown of Bmi1 increases sensitivity of breast cells to doxorubicin and reduced tumorigenicity

**Figure 4:**
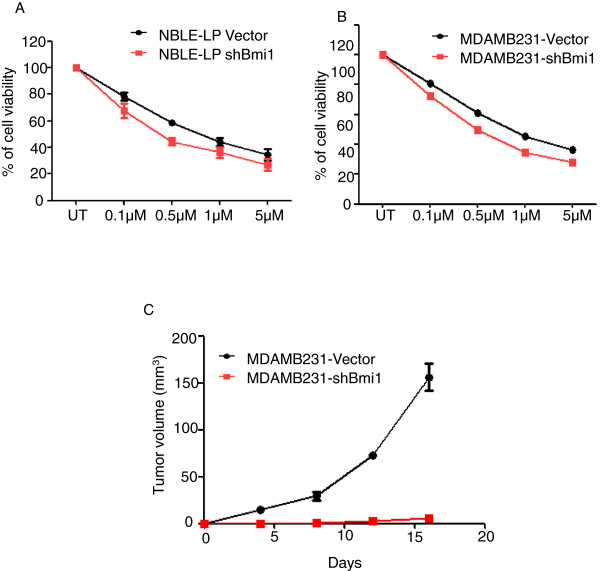
**Knockdown of Bmi1 increased sensitivity of cells to cytotoxic drug doxorubicin and reduced tumorigenicity.** The graphs show the dose–response curve of NBLE-LP **(A)** and MDAMB231 **(B)** cells with vector alone control or shBmi1, treated with doxorubicin for 48 hrs (n =3). **C**. The chart shows number of tumors formed by MDAMB231 cells with vector alone control or with shBmi1.

RT-PCR analysis indicated that overexpression of Bmi1 resulted in increased expression of ABC transporters such as ABCG2 and ABCC1 expression (Figure 2C and D) that mediate drug-resistance through membrane bound efflux pumps [42]. Few studies have associated Bmi1 with radio-resistance and drug-resistance in breast cancer cells [43, 44]. To study the effect of Bmi1 knockdown on drug response of NBLE-LP and MDAMB231 cells we treated vector control and shBmi1 cells with anti-cancer drug doxorubicin for 48 hrs. As shown in the dose–response curves, knockdown of Bmi1 increased their chemosensitivity by 55% in NBLE-LP cells and by 57% in MDAMB231 cells, as evident by decreased IC_50_ values (Figure 4A, B, and Additional file 1: Figure S5) [IC_50_ (μM): NBLE-LP-Vector: 0.8534, NBLE-LP-shBmi1: 0.4674, MDAMB231-Vector: 0.8084, and MDAMB231-shBmi1: 0.3955]. This observation was similar to an earlier study in which knockdown of Bmi1 in MCF7 reduced the IC_50_ from 0.87 to 0.15 (μg/ml) [43]. Thus, these data indicated that reduction of Bmi1 expression leads to considerable increase in sensitivity of cancer cells to drugs, and might thus help in cancer chemotherapy.

Our results thus far implied that Bmi1 plays a crucial role in self-renewal, EMT and drug-resistance of breast cancer cells - the properties attributed to tumor-initiating cells [45]. Thus, we investigated if knockdown of Bmi1 had any effect on tumor initiation in xenograft models. We injected 1×10^6^ MDAMB231-Vector and shBmi1 cells in either flanks of nude mice. We observed that Bmi1 knockdown abrogated tumor formation (Figure 4C). Our results thus confirmed that knockdown of Bmi1 rendered the cells sensitive to chemotherapeutic drugs and revealed that Bmi1 is necessary for tumorigenicity of breast cancer cells.

### Bmi1 positively regulates Nanog expression in mammary epithelial cells

**Figure 5:**
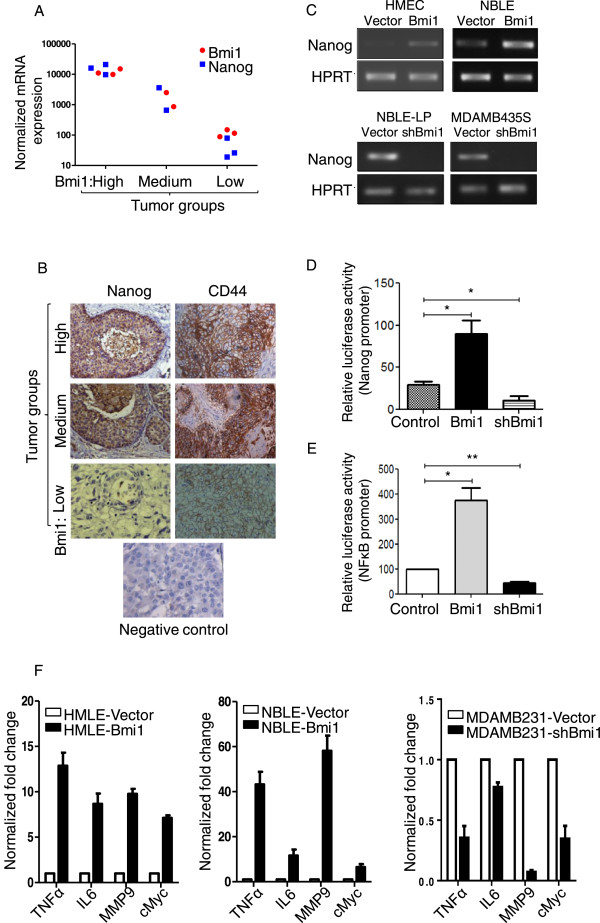
**Bmi1 positively regulates Nanog expression in mammary epithelial cells. A**. Graph showing normalized mRNA levels (in arbitrary units) of Bmi1 and Nanog in primary breast tissues. X-axis shows tumors categorized into Bmi1-high, moderate, and low groups. **B**. The images show immunohistochemical staining for Nanog and CD44 on tumor tissues that showed high, medium and low Bmi1 levels (also refer to Figure 1C). **C**. The pictographs show Nanog levels assessed by RT-PCR in indicated cell types (HMEC-primary normal human mammary epithelial cells). **D**. Graph represents luciferase assay in HEK293T cells to assess the Nanog promoter activity (pGL3-Nanog-Luc) on co-transfection with pRL-TK (Renilla luciferase), pLKO1, pBABEpuro-Bmi1 and pLKO1-shBmi1 vectors. After 48 hrs of culturing Firefly luciferase activity was measured and normalized to Renilla luciferase activity and represented as relative luciferase units (RLU), n =3; error bars indicate s.d, (statistical significance was calculated using unpaired *t*-test between relative luciferase activity between vector control and Bmi1 overexpression; and between vector control and Bmi1 knockdown, * = p <0.05). **E**. Graph represents luciferase assay to assess the NFκB promoter activity in HEK293T cells upon Bmi1 overexpression and Bmi1 knockdown compared to the vector (pLKO1) alone cells. After 48 hrs of culturing Firefly luciferase activity was measured and normalized to Renilla luciferase activity and represented as relative luciferase units (RLU), n =3; error bars indicate s.d, (statistical significance was calculated using unpaired *t*-test between relative luciferase activity between vector control and Bmi1 overexpression; and between vector control and Bmi1 knockdown, * = p <0.05, ** = p <0.01). **F**. Graphs show fold change of NFκB pathway genes (normalized to β2M mRNA levels) with control vector or Bmi1 overexpression or down-modulation in HMLE, NBLE, and MDAMB231 cells, n =3; error bars indicate s.d).

In various primary breast tumors analyzed by RT-PCR, we found that the tumors with higher Bmi1 also showed elevated Nanog and the tumors with lower Bmi1 showed reduced Nanog expression (Figure 5A). Immunohistochemical analysis also revealed similar results. Interestingly, CD44 expression also varied similarly (Figure 5B). Furthermore, overexpression of Bmi1 in primary HMECs and NBLE cells led to an increased expression of Nanog (Figure 5C), while knockdown of Bmi1 in NBLE-LP and MDAMB231 cells led to a reduction in Nanog expression (Figure 5C, also refer to Figures 2E and 3E). Together, these data suggested that Bmi1 might regulate Nanog expression in mammary epithelial cells.

To further investigate if Bmi1 regulated Nanog expression, we made use of a promoter reporter assay using dual luciferase assay. We used a luciferase construct downstream of Nanog promoter (a kind gift from Dr. Jyotsna Dhawan, InStem, Bangalore and Dr. Takashi Tada, Kyoto University, Kyoto, Japan) to assess the effect of Bmi1 on Nanog promoter activity in HEK293T cells expressing Bmi1 or shBmi1. Compared to parental cells, those overexpressing Bmi1 showed increased Nanog promoter activity while those with shBmi1 showed reduced Nanog promoter activity (Figure 5D). These results indicated that Bmi1 might positively regulate Nanog expression.

Bmi1 has been shown to increase angiogenesis, tumor aggressiveness, and resistance to apoptosis in glioma cells by activating the NFκB pathway [18, 46, 47]. Recent studies have suggested a major role for NFκB pathway in mammary stem cell self-renewal [48]. Another study showed that in mammary epithelial cells, NFκB regulated Nanog expression [49]. In that study, activation of NFκB through p65/p50 increased Nanog promoter activity and inhibition of NFκB through IκBα super repressor (IκBαSR) reduced the Nanog promoter activity significantly. Since in our study we observed that Bmi1 overexpression led to an increase, while its knockdown led to reduced Nanog promoter activity, we asked if Bmi1 mediates its effects through regulating the NFkB pathway. As assessed by NFκB promoter reporter assay we observed that HEK293T cells overexpressing Bmi1 showed increased NFκB promoter activity, whereas upon Bmi1 knockdown the NFκB promoter activity was reduced compared to the control cells (Figure 5E). By RT-PCR we analyzed mRNA levels of various NFκB pathway targets including TNFα, IL6, MMP9, and cMyc. We observed that overexpression of Bmi1 increased the mRNA levels of NFκB targets whereas Bmi1 knockdown reduced their expression (Figure 5F). Taken together, our data suggested that Bmi1 regulates the stemness properties of breast cancer cells, at least in part, via regulating the expression of Nanog through the NFκB pathway.

## Discussion

Bmi1 was first identified as a c-Myc cooperating oncogene in lymphomagenesis [14, 20]. Since then, Bmi1 has been associated with several cancers including non-small cell lung cancer [22], ovarian cancer [23], acute myeloid leukemia [50], nasopharyngeal carcinoma [51], neuroblastoma [52], glioblastoma [53], and breast cancer [24]. Bmi1 mRNA was found to be higher in the plasma of breast cancer patients compared to normal sample [24]. This suggested Bmi1 could be used as a prognostic marker in breast cancer patients. More recently, Bmi1 has been shown to be involved with the regulation of stem cell self-renewal. In this study we show that Bmi1 upregulation is associated with grade III invasive ductal adenocarcinomas of Indian patients, further supporting its role as a prognostic marker. We further show that Bmi1 expression is involved with the regulation of stemness associated genes, and show that one possible mechanism by which Bmi1 may regulate stemness and self-renewal is by the upregulation of Nanog, a stemness regulating gene, through the NFκB pathway.

Few studies also found a correlation between Bmi1 expression and lymph node metastasis in breast cancer [54, 55] suggesting a role for Bmi1 in cancer metastasis. This observation was further corroborated with a report that showed Bmi1 promoted invasion and metastasis in breast cancer and predicted poor survival [16]. Consistent with this, we show here that Bmi1 overexpression induced EMT, a process closely associated with breast cancer metastasis [56], while its down-modulation reversed EMT. These observations suggest that targeting Bmi1 could be employed in treating breast cancer metastasis. Preclinical and clinical evidences suggested that Bmi1 mediates chemoresistance in various cancers [57]. We observed that knockdown of Bmi1 rendered NBLE and MDAMB231 cells more sensitive to doxorubicin, a commonly used chemotherapeutic agent. Bmi1 might be aiding in drug-resistance partly through ABC transporters, as Bmi1 knockdown reduced expression of ABCC1 and ABCG2 expression. Hence, Bmi1 might be an ideal target to overcome chemoresistance and tumor relapse.

Recent developments that attribute key role to ‘cancer stem cells’ in tumor initiation and progression demand a greater probe into understanding molecular pathways regulating self-renewal in these cells. Many studies have now conclusively shown that Polycomb proteins and in particular Bmi1 play critical role in regulating self-renewal and proliferation of both normal and cancer stem cells of various tissues [13]. In mice, loss of Bmi1 resulted in acute mammary epithelial growth defects and Bmi1 was necessary for proliferation of epithelial cells and to prevent premature differentiation [58]. Another study reported that Hedgehog signaling regulates self-renewal of both normal and tumorigenic human mammary stem/progenitor cells through Bmi1 [34]. It has been speculated that Bmi1 might be regulated also by other self-renewal pathways such as Notch and Wnt [13]. If Bmi1 is a key player responsible for the crosstalk between these self-renewal pathways, targeting Bmi1 could be an ideal way of targeting these pathways that are activated in breast cancer stem cells [59]. In support of this we observed that overexpression of Bmi1 resulted in increased expression of Notch, Wnt, and Hh pathway players such as Hes1, Hes5, Axin2, Lef1, Gli1 and Gli2 whereas Bmi1 knockdown reduced the expression of these genes. It would be interesting to further understand how the complex interplay between these self-renewal pathways are orchestrated with Bmi1 being the conductor.

Even though Bmi1’s role has been clearly established in stem cells, no studies have yet conclusively shown the mechanism by which Bmi1 regulates stem cell self-renewal. Our novel finding that Bmi1 might upregulate the expression of Nanog, a known embryonic stem cell marker, through activating the NFκB pathway might shed light on understanding the mechanisms downstream of Bmi1 in the regulation of stem cell self-renewal. NFκB pathway has been shown to be involved in proliferation and branching of mammary epithelial cells during post-natal development [60]. It was also reported that activation of NFκB pathway helps in increased proliferation and resistance to apoptosis in distinct subclass of ER-negative breast cancers [61]. Further, NFκB was shown to govern tumor stem cell expansion in a transgenic mouse model [49], and IκB kinase was shown to be necessary for self-renewal of Her2-transformed mammary tumor initiating cells [62], thus highlighting the significance of NFκB pathway in breast cancer stem cell self-renewal [48]. Although Nanog is not expressed in most adult tissues, it was reported that Nanog is expressed in various cancers including breast cancer [63–67]. A recent study showed that expression of stemness transcription factors including Nanog correlated with the stage of the breast cancer [68]. Nanog overexpression along with Wnt1 in mouse mammary gland resulted in mammary tumorigenesis and metastasis and promoted the migration and invasion of breast cancer cells [67]. Our observation that Bmi1 and Nanog are co-expressed in breast tumors, and importantly that Bmi1 positively regulated Nanog expression, at least in part, by activating NFκB pathway helps in connecting the missing link. Further studies on Bmi1 mediated regulation of NFκB pathway and Nanog expression in cancer and cancer stem cells would immensely help in understanding the molecular pathways that initiate, drive, and maintain these tumors.

## Conclusions

To conclude, our study showed that Bmi1 is upregulated in primary breast tumors. Overexpression of Bmi1 in mammary epithelial cells increased self-renewal and stemness, and induced mesenchymal morphology with increase in the expression of EMT-related genes. These observations were reversed on Bmi1 knockdown. Further, reduced Bmi1 rendered breast cancer cells increasingly susceptible to chemotherapeutic drug doxorubicin and abrogated tumor formation in nude mice. Dual-luciferase assay showed that Bmi1 positively regulates a known stem cell marker Nanog via NFκB pathway. This study provides novel insights into various roles of Bmi1 in breast cancer and indicates that targeting Bmi1 could inhibit cancer stem cells and revert EMT. Further studies on these novel observations would help in identifying proteins that can be targeted in cancer stem cell-specific therapy and hold a great promise in breast cancer therapeutics.

## Electronic supplementary material

Additional file 1: Figure S1: The sequence of the primers used in the study. **Figure S2.** Nucleotide sequence of Bmi1 shRNA used in the study. **Figure S3.** The status of Bmi1 protein levels in 25 primary breast adenocarcinoma samples as analyzed by immunohistochemistry. High:+++, Medium:++,Low:+. **Figure S4.** Representative images showing morphology of NBLE-LP and MDAMB231 cells with vector alone or upon Bmi1knockdown. **Figure S5.** Non-linear regression curve (Curvefit) to calculate IC_50_ value of doxorubicin in NBLE-LP-Vector and NBLE-LP-shBmi1 stable cell lines (A) and MDAMB231-Vector and MDAMB231-shBmi1 stable cell lines (B); n = 3, error bars indicate s.d. (PDF 325 KB)

## Abbreviations

BCSC: Breast cancer stem cells
Bmi1: B lymphoma Mo-MLV insertion region 1 homolog
EMT: Epithelial to mesenchymal transition
IHC: Immunohistochemistry
LP: Late passage
NB: Normal breast.

## References

[1] Pardal R, Clarke MF, Morrison SJ (2003). Applying the principles of stem-cell biology to cancer. Nat Rev Cancer.

[2] Lapidot T, Sirard C, Vormoor J, Murdoch B, Hoang T, Caceres-Cortes J, Minden M, Paterson B, Caligiuri MA, Dick JE (1994). A cell initiating human acute myeloid leukaemia after transplantation into SCID mice. Nature.

[3] Bonnet D, Dick JE (1997). Human acute myeloid leukemia is organized as a hierarchy that originates from a primitive hematopoietic cell. Nat Med.

[4] Visvader JE, Lindeman GJ (2008). Cancer stem cells in solid tumours: accumulating evidence and unresolved questions. Nat Rev Cancer.

[5] Singh SK, Clarke ID, Terasaki M, Bonn VE, Hawkins C, Squire J, Dirks PB (2003). Identification of a cancer stem cell in human brain tumors. Cancer Res.

[6] Li C, Heidt DG, Dalerba P, Burant CF, Zhang L, Adsay V, Wicha M, Clarke MF, Simeone DM (2007). Identification of pancreatic cancer stem cells. Cancer Res.

[7] O’Brien CA, Pollett A, Gallinger S, Dick JE (2007). A human colon cancer cell capable of initiating tumour growth in immunodeficient mice. Nature.

[8] Al-Hajj M, Wicha MS, Benito-Hernandez A, Morrison SJ, Clarke MF (2003). Prospective identification of tumorigenic breast cancer cells. Proc Natl Acad Sci U S A.

[9] Zhou BB, Zhang H, Damelin M, Geles KG, Grindley JC, Dirks PB (2009). Tumour-initiating cells: challenges and opportunities for anticancer drug discovery. Nat Rev Drug Discov.

[10] Mani SA, Guo W, Liao MJ, Eaton EN, Ayyanan A, Zhou AY, Brooks M, Reinhard F, Zhang CC, Shipitsin M, Campbell LL, Polyak K, Brisken C, Yang J, Weinberg RA (2008). The epithelial-mesenchymal transition generates cells with properties of stem cells. Cell.

[11] Saxena M, Stephens MA, Pathak H, Rangarajan A (2011). Transcription factors that mediate epithelial-mesenchymal transition lead to multidrug resistance by upregulating ABC transporters. Cell Death Dis.

[12] Singh A, Settleman J (2010). EMT, cancer stem cells and drug resistance: an emerging axis of evil in the war on cancer. Oncogene.

[13] Valk-Lingbeek ME, Bruggeman SW, van Lohuizen M (2004). Stem cells and cancer; the polycomb connection. Cell.

[14] van Lohuizen M, Verbeek S, Scheijen B, Wientjens E, van der Gulden H, Berns A (1991). Identification of cooperating oncogenes in E mu-myc transgenic mice by provirus tagging. Cell.

[15] Yang MH, Hsu DS, Wang HW, Wang HJ, Lan HY, Yang WH, Huang CH, Kao SY, Tzeng CH, Tai SK, Chang SY, Lee OK, Wu KJ (2010). Bmi1 is essential in Twist1-induced epithelial-mesenchymal transition. Nat Cell Biol.

[16] Guo BH, Feng Y, Zhang R, Xu LH, Li MZ, Kung HF, Song LB, Zeng MS (2011). Bmi-1 promotes invasion and metastasis, and its elevated expression is correlated with an advanced stage of breast cancer. Mol Cancer.

[17] Dimri GP, Martinez JL, Jacobs JJ, Keblusek P, Itahana K, Van Lohuizen M, Campisi J, Wazer DE, Band V (2002). The Bmi-1 oncogene induces telomerase activity and immortalizes human mammary epithelial cells. Cancer Res.

[18] Jiang L, Wu J, Yang Y, Liu L, Song L, Li J, Li M (2012). Bmi-1 promotes the aggressiveness of glioma via activating the NF-kappaB/MMP-9 signaling pathway. BMC Cancer.

[19] Simon JA, Kingston RE (2009). Mechanisms of polycomb gene silencing: knowns and unknowns. Nat Rev Mol Cell Biol.

[20] Jacobs JJ, Scheijen B, Voncken JW, Kieboom K, Berns A, van Lohuizen M (1999). Bmi-1 collaborates with c-Myc in tumorigenesis by inhibiting c-Myc-induced apoptosis via INK4a/ARF. Genes Dev.

[21] Jacobs JJ, Kieboom K, Marino S, DePinho RA, van Lohuizen M (1999). The oncogene and Polycomb-group gene bmi-1 regulates cell proliferation and senescence through the ink4a locus. Nature.

[22] Vonlanthen S, Heighway J, Altermatt HJ, Gugger M, Kappeler A, Borner MM, van Lohuizen M, Betticher DC (2001). The bmi-1 oncoprotein is differentially expressed in non-small cell lung cancer and correlates with INK4A-ARF locus expression. Br J Cancer.

[23] Zhang F, Sui L, Xin T (2008). Correlations of BMI-1 expression and telomerase activity in ovarian cancer tissues. Exp Oncol.

[24] Silva J, Garcia V, Garcia JM, Pena C, Dominguez G, Diaz R, Lorenzo Y, Hurtado A, Sanchez A, Bonilla F (2007). Circulating Bmi-1 mRNA as a possible prognostic factor for advanced breast cancer patients. Breast Cancer Res.

[25] Kohler C, Villar CB (2008). Programming of gene expression by polycomb group proteins. Trends Cell Biol.

[26] Song LB, Li J, Liao WT, Feng Y, Yu CP, Hu LJ, Kong QL, Xu LH, Zhang X, Liu WL, Li MZ, Zhang L, Kang TB, Fu LW, Huang WL, Xia YF, Tsao SW, Li M, Band V, Band H, Shi QH, Zeng YX, Zeng MS (2009). The polycomb group protein Bmi-1 represses the tumor suppressor PTEN and induces epithelial-mesenchymal transition in human nasopharyngeal epithelial cells. J Clin Invest.

[27] Schuringa JJ, Vellenga E (2010). Role of the polycomb group gene BMI1 in normal and leukemic hematopoietic stem and progenitor cells. Curr Opin Hematol.

[28] Lessard J, Sauvageau G (2003). Bmi-1 determines the proliferative capacity of normal and leukaemic stem cells. Nature.

[29] Molofsky AV, Pardal R, Iwashita T, Park IK, Clarke MF, Morrison SJ (2003). Bmi-1 dependence distinguishes neural stem cell self-renewal from progenitor proliferation. Nature.

[30] Godlewski J, Nowicki MO, Bronisz A, Williams S, Otsuki A, Nuovo G, Raychaudhury A, Newton HB, Chiocca EA, Lawler S (2008). Targeting of the Bmi-1 oncogene/stem cell renewal factor by microRNA-128 inhibits glioma proliferation and self-renewal. Cancer Res.

[31] Chiba T, Miyagi S, Saraya A, Aoki R, Seki A, Morita Y, Yonemitsu Y, Yokosuka O, Taniguchi H, Nakauchi H, Iwama A (2008). The polycomb gene product BMI1 contributes to the maintenance of tumor-initiating side population cells in hepatocellular carcinoma. Cancer Res.

[32] Lukacs RU, Memarzadeh S, Wu H, Witte ON (2010). Bmi-1 is a crucial regulator of prostate stem cell self-renewal and malignant transformation. Cell Stem Cell.

[33] Proctor E, Waghray M, Lee CJ, Heidt DG, Yalamanchili M, Li C, Bednar F, Simeone DM (2013). Bmi1 enhances tumorigenicity and cancer stem cell function in pancreatic adenocarcinoma. PLoS One.

[34] Liu S, Dontu G, Mantle ID, Patel S, Ahn NS, Jackson KW, Suri P, Wicha MS (2006). Hedgehog signaling and Bmi-1 regulate self-renewal of normal and malignant human mammary stem cells. Cancer Res.

[35] Mittal S, Subramanyam D, Dey D, Kumar RV, Rangarajan A (2009). Cooperation of Notch and Ras/MAPK signaling pathways in human breast carcinogenesis. Mol Cancer.

[36] Paranjape AN, Mandal T, Mukherjee G, Kumar MV, Sengupta K, Rangarajan A (2012). Introduction of SV40ER and hTERT into mammospheres generates breast cancer cells with stem cell properties. Oncogene.

[37] Yuan B, Latek R, Hossbach M, Tuschl T, Lewitter F (2004). siRNA Selection Server: an automated siRNA oligonucleotide prediction server. Nucleic Acids Res.

[38] Stewart SA, Dykxhoorn DM, Palliser D, Mizuno H, Yu EY, An DS, Sabatini DM, Chen IS, Hahn WC, Sharp PA, Weinberg RA, Novina CD (2003). Lentivirus-delivered stable gene silencing by RNAi in primary cells. RNA.

[39] Cao L, Bombard J, Cintron K, Sheedy J, Weetall ML, Davis TW (2011). BMI1 as a novel target for drug discovery in cancer. J Cell Biochem.

[40] Elenbaas B, Spirio L, Koerner F, Fleming MD, Zimonjic DB, Donaher JL, Popescu NC, Hahn WC, Weinberg RA (2001). Human breast cancer cells generated by oncogenic transformation of primary mammary epithelial cells. Genes Dev.

[41] Dontu G, Abdallah WM, Foley JM, Jackson KW, Clarke MF, Kawamura MJ, Wicha MS (2003). *In vitro* propagation and transcriptional profiling of human mammary stem/progenitor cells. Genes Dev.

[42] Dean M (2009). ABC transporters, drug resistance, and cancer stem cells. J Mammary Gland Biol Neoplasia.

[43] Wu X, Liu X, Sengupta J, Bu Y, Yi F, Wang C, Shi Y, Zhu Y, Jiao Q, Song F (2011). Silencing of Bmi-1 gene by RNA interference enhances sensitivity to doxorubicin in breast cancer cells. Indian J Exp Biol.

[44] Liu ZG, Liu L, Xu LH, Yi W, Tao YL, Tu ZW, Li MZ, Zeng MS, Xia YF (2012). Bmi-1 induces radioresistance in MCF-7 mammary carcinoma cells. Oncol Rep.

[45] Nicolini A, Ferrari P, Fini M, Borsari V, Fallahi P, Antonelli A, Berti P, Carpi A, Miccoli P (2011). Stem cells: their role in breast cancer development and resistance to treatment. Curr Pharm Biotechnol.

[46] Jiang L, Song L, Wu J, Yang Y, Zhu X, Hu B, Cheng SY, Li M (2013). Bmi-1 promotes glioma angiogenesis by activating NF-kappaB signaling. PLoS One.

[47] Li J, Gong LY, Song LB, Jiang LL, Liu LP, Wu J, Yuan J, Cai JC, He M, Wang L, Zeng M, Cheng SY, Li M (2010). Oncoprotein Bmi-1 renders apoptotic resistance to glioma cells through activation of the IKK-nuclear factor-kappaB Pathway. Am J Pathol.

[48] Shostak K, Chariot A (2011). NF-kappaB, stem cells and breast cancer: the links get stronger. Breast Cancer Res.

[49] Liu M, Sakamaki T, Casimiro MC, Willmarth NE, Quong AA, Ju X, Ojeifo J, Jiao X, Yeow WS, Katiyar S, Shirley LA, Joyce D, Lisanti MP, Albanese C, Pestell RG (2010). The canonical NF-kappaB pathway governs mammary tumorigenesis in transgenic mice and tumor stem cell expansion. Cancer research.

[50] Sawa M, Yamamoto K, Yokozawa T, Kiyoi H, Hishida A, Kajiguchi T, Seto M, Kohno A, Kitamura K, Itoh Y, Asou N, Hamajima N, Emi N, Naoe T (2005). BMI-1 is highly expressed in M0-subtype acute myeloid leukemia. Int J Hematol.

[51] Song LB, Zeng MS, Liao WT, Zhang L, Mo HY, Liu WL, Shao JY, Wu QL, Li MZ, Xia YF, Fu LW, Huang WL, Dimri GP, Band V, Zeng YX (2006). Bmi-1 is a novel molecular marker of nasopharyngeal carcinoma progression and immortalizes primary human nasopharyngeal epithelial cells. Cancer research.

[52] Cui H, Hu B, Li T, Ma J, Alam G, Gunning WT, Ding HF (2007). Bmi-1 is essential for the tumorigenicity of neuroblastoma cells. Am J Pathol.

[53] Cenci T, Martini M, Montano N, D'Alessandris QG, Falchetti ML, Annibali D, Savino M, Bianchi F, Pierconti F, Nasi S, Pallini R, Larocca LM (2012). Prognostic relevance of c-Myc and BMI1 expression in patients with glioblastoma. Am J Clin Pathol.

[54] Kim JH, Yoon SY, Jeong SH, Kim SY, Moon SK, Joo JH, Lee Y, Choe IS, Kim JW (2004). Overexpression of Bmi-1 oncoprotein correlates with axillary lymph node metastases in invasive ductal breast cancer. Breast.

[55] Gavrilescu MM, Todosi AM, Anitei MG, Filip B, Scripcariu V (2012). Expression of bmi-1 protein in cervical, breast and ovarian cancer. Rev Med Chir Soc Med Nat Iasi.

[56] Wang Y, Zhou BP (2011). Epithelial-mesenchymal transition in breast cancer progression and metastasis. Chin J Cancer.

[57] Siddique HR, Saleem M (2012). Role of BMI1, a stem cell factor, in cancer recurrence and chemoresistance: preclinical and clinical evidences. Stem Cells.

[58] Pietersen AM, Evers B, Prasad AA, Tanger E, Cornelissen-Steijger P, Jonkers J, van Lohuizen M (2008). Bmi1 regulates stem cells and proliferation and differentiation of committed cells in mammary epithelium. Curr Biol.

[59] Liu S, Dontu G, Wicha MS (2005). Mammary stem cells, self-renewal pathways, and carcinogenesis. Breast Cancer Res.

[60] Brantley DM, Chen CL, Muraoka RS, Bushdid PB, Bradberry JL, Kittrell F, Medina D, Matrisian LM, Kerr LD, Yull FE (2001). Nuclear factor-kappaB (NF-kappaB) regulates proliferation and branching in mouse mammary epithelium. Mol Biol Cell.

[61] Biswas DK, Shi Q, Baily S, Strickland I, Ghosh S, Pardee AB, Iglehart JD (2004). NF-kappa B activation in human breast cancer specimens and its role in cell proliferation and apoptosis. Proc Natl Acad Sci U S A.

[62] Cao Y, Luo JL, Karin M (2007). IkappaB kinase alpha kinase activity is required for self-renewal of ErbB2/Her2-transformed mammary tumor-initiating cells. Proc Natl Acad Sci U S A.

[63] Almstrup K, Hoei-Hansen CE, Wirkner U, Blake J, Schwager C, Ansorge W, Nielsen JE, Skakkebaek NE, Rajpert-De Meyts E, Leffers H (2004). Embryonic stem cell-like features of testicular carcinoma *in situ* revealed by genome-wide gene expression profiling. Cancer Res.

[64] Ezeh UI, Turek PJ, Reijo RA, Clark AT (2005). Human embryonic stem cell genes OCT4, NANOG, STELLAR, and GDF3 are expressed in both seminoma and breast carcinoma. Cancer.

[65] Hart AH, Hartley L, Parker K, Ibrahim M, Looijenga LH, Pauchnik M, Chow CW, Robb L (2005). The pluripotency homeobox gene NANOG is expressed in human germ cell tumors. Cancer.

[66] Chiou SH, Wang ML, Chou YT, Chen CJ, Hong CF, Hsieh WJ, Chang HT, Chen YS, Lin TW, Hsu HS, Wu CW (2010). Coexpression of Oct4 and nanog enhances malignancy in lung adenocarcinoma by inducing cancer stem cell-like properties and epithelial-mesenchymal transdifferentiation. Cancer Res.

[67] Lu X, Mazur SJ, Lin T, Appella E, Xu Y (2013). The pluripotency factor nanog promotes breast cancer tumorigenesis and metastasis. Oncogene.

[68] Apostolou P, Toloudi M, Chatziioannou M, Ioannou E, Papasotiriou I (2012). Cancer stem cells stemness transcription factors expression correlates with breast cancer disease stage. Curr Stem Cell Res Ther.

[CR69] The pre-publication history for this paper can be accessed here:http://www.biomedcentral.com/1471-2407/14/785/prepub

