# Supplementary material for: Bmi1 regulates self-renewal and epithelial to mesenchymal transition in breast cancer cells through Nanog
Source: BMC Cancer. 2014 Oct 28;14:785. doi: 10.1186/1471-2407-14-785 (PMC4223733; doi:10.1186/1471-2407-14-785)
Supplement: Supplementary file 1 — Additional file 1: Figure S1: The sequence of the primers used in the study. Figure S2. Nucleotide sequence of Bmi1 shRNA used in the study. Figure S3. The status of Bmi1 protein levels in 25 primary breast adenocarcinoma samples as analyzed by immunohistochemistry. High:+++, Medium:++,Low:+. Figure S4. Representative images showing morphology of NBLE-LP and MDAMB231 cells with vector alone or upon Bmi1knockdown. Figure S5. Non-linear regression curve (Curvefit) to calculate IC50 value of doxorubicin in NBLE-LP-Vector and NBLE-LP-shBmi1 stable cell lines (A) and MDAMB231-Vector and MDAMB231-shBmi1 stable cell lines (B); n = 3, error bars indicate s.d. (PDF 325 KB) [file 12885_2014_4966_MOESM1_ESM.pdf]

Supplementary Figure 1

Gene name	Primer sequence
ABCC1-FW	5’-AGCCGGTGAAGGTTGTGTAC -3’
ABCC1-RV	5’-TGACGAAGCAGATGTGGAAG -3’
ABCG2-FW	5’-GCAGATGCCTTCTTCGTTATG -3’
ABCG2-RV	5’-TCTTCGCCAGTACATGTTGC -3’
AXIN2-FW	5’-CCAGACTCAGTGGGAAGAGC -3’
AXIN2-RV	5’- AAGAGACAGGCATGGGTTTG -3’
BMI1-FW	5’- CCAGGGCTTTTCAAAAATGA -3’
BMI1-RV	5’-CCGATCCAATCTGTTCTGGT -3’
CD44 -FW	5’- GGGATTGGTTTTCATGGTTG -3’
CD44 -RV	5’-GTGTGGTTGAAATGGTGCTG -3’
Gli-1-FW	5’ –GTGCAAGTCAAGCCAGAACA -3’
Gli-1-RV	5’-ATAGGGGCCTGACTGGAGAT -3’
Gli-2-FW	5’-TGGAATTTGGAACTGGCTTC -3’
Gli-2-RV	5’-CCTCATTAAGGCCAAGGTCA -3’
Hes-1-FW	5’-TTCCCTCCGGACTCTAAACA -3’
Hes-1-RV	5’-CAAACATCTTTGGCATCACA -3’
Hes-5-FW	5’- GCCGTTTTAGGACAATCAGG -3’
Hes-5-RV	5’-GAGTTCGGCCTTCACAAAAG -3’
HPRT-FW	5’- TGCTCGAGATGTGATGAAGG -3’
HPRT-RV	5’- TCCCCTGTTGACTGGTCATT -3’
Nanog-FW	5’- CCTCCTCCATGGATCTGCTTATTCA -3’
Nanog-RV	5’-CAGGTCTTCACCTGTTTGTAG -3’
N-Cadherin-FW	5’-GACAATGCCCCTCAAGTGTT -3’
N-Cadherin-RV	5’-CCATTAAGCCGAGTGATGGT -3’
Oct4-FW	5’-CGACCATCTGCCGCTTTGAG-3’
Oct4-RW	5’- CCCCCTGTCCCCCATTCCTA -3’
RPL-FW	5'-TTAAATCCTTGAGGGGTACA-3'
RPL-RV	5'-ACGGGACTTCTAAAAGGAAC-3'
Sox2-FW	5’-CCCCCGGCGGCAATAGCA -3’
Sox2-RV	5’-TCGGCGCCGGGGACATACAT -3’
Twist1-FW	5’-GTCCGCAGTCTTACGAGGAG -3’
Twist1-RV	5’-CCAGCTTGAGGGTCTGAATC -3’
Vimentin-FW	5’-GAGAACTTTGCCGTTGAAGC -3’
Vimentin-RV	5’-TCCAGCAGCTTCCTGTAGGT -3’

Supplementary Figure 1.The sequence of the primers used in the study.

shBmi1- Sense:
CCGGAACCAGACCACTACTGAATATCTCGAGATATTCAGTAGTGGTCTG
GTTTTTTTG

shBmi1- Anti Sense:
AATTCAAAAAAACCAGACCACTACTGAATATCTCGAGATATTCAGTAGT
GGTCTGGTT

Supplementary Figure 2. Nucleotide sequence of Bmi1 shRNA used in the study.

Supplementary Figure 3

Sl No.	Tissue No.	Bmi1 intensity
1	BT101	+
2	BT102	+
3	BT103	+
4	BT104	+
5	BT105	+
6	BT106	+
7	BT107	+
8	BT108	+
9	BT109	+
10	BT110	++
11	BT111	++
12	BT112	++
13	BT113	++
14	BT114	++
15	BT115	++
16	BT116	++
17	BT117	++
18	BT118	++
19	BT119	++
20	BT120	++
21	BT121	+++
22	BT122	+++
23	BT123	+++
24	BT124	+++
25	BT125	+++

Supplementary Figure 3. The status of Bmi1 protein levels in 25 primary breast adenocarcinoma samples as analyzed by immunohistochemistry. High: +++, Medium: ++, Low: +.

Supplementary Figure 4

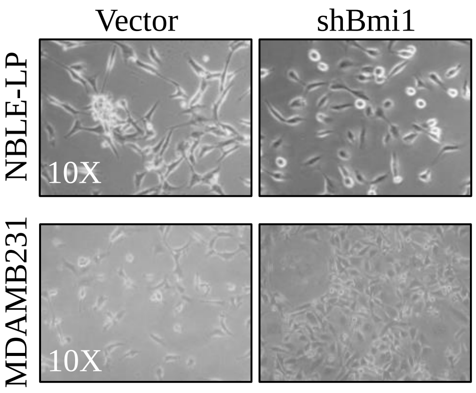

Supplementary Figure 4. Representative images showing morphology of NBLE-LP and MDAMB231 cells with vector alone or upon Bmi1 knockdown.

Supplementary Figure 5

A

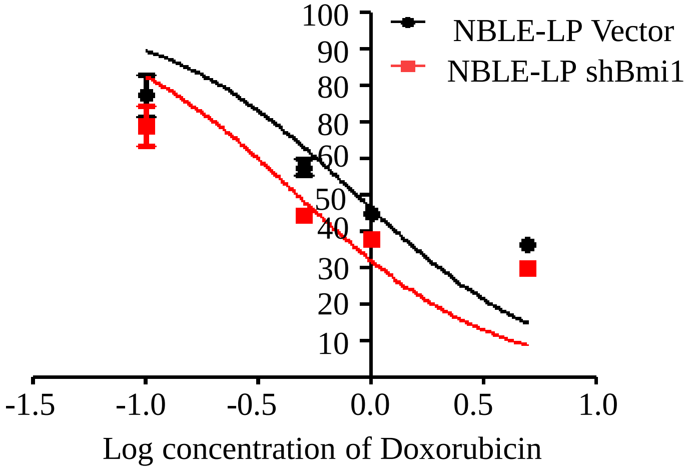

	NBLE-LP Vector	NBLE-LP shBmi1
IC_{50}	0.8534	0.4674

B

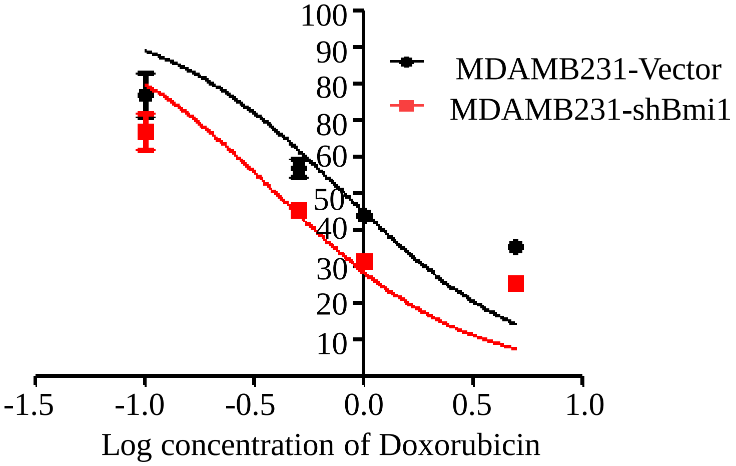

	MDAMB231-Vector	MDAMB231-shBmi1
IC_{50}	0.8084	0.3955

Supplementary Figure 5. Non-linear regression curve (Curve fit) to calculate IC_{50} value of doxorubicin in NBLE-LP vector and NBLE-LP shBmi1 stable cell lines (A) and MDAMB231-Vector and MDAMB231-shBmi1 stable cell lines (B); n=3, error bars indicate S.D.
